# Undifferentiated Shock and Extreme Elevation of Procalcitonin Related to Kratom Use

**DOI:** 10.5005/jp-journals-10071-23170

**Published:** 2019-05

**Authors:** Muhammad Zuberi, Pramod Kumar Guru, Vikas Bansal, Jose Diaz-Gomez, Beth Grieninger, David Alejos

**Affiliations:** 1-6 Department of Critical Care Medicine, Mayo Clinic, Florida, USA

**Keywords:** Kratom, Procalcitonin, Vasopressors

## Abstract

An 18-year-old male with history of polysubstance abuse presented to the emergency department with intractable vomiting, diarrhea, and abdominal pain for one day after the consumption of kratom. Examination revealed arterial hypotension, tachycardia, and prolonged capillary refill. Laboratory studies showed white blood cell count (WBC) of 23.6 × 109/L, serum creatinine 4.0 mg/dL, lactate 6 mmol/L, and procalcitonin >200 ng/mL. Urine and blood drug screen were unremarkable. Radiology and echocardiogram were noncontributory. He received fluid resuscitation and broad spectrum antibiotics. Vasopressors were subsequently added to manage persistent shock. He remained afebrile, and his blood cultures were negative. His shock and associated organ dysfunctions improved over the next 72 hours. On discharge, his procalcitonin level decreased to 9.55 ng/mL, leukocytosis resolved, and the creatinine returned to baseline. This case describes an extremely rare presentation related to kratom, an herb marketed as an opioid alternative, with significant potential for addiction and withdrawal syndrome.

**How to cite this article:** Zuberi M, Guru PK, Bansal V, Diaz-Gomez J, Grieninger B, Alejos D. Undifferentiated Shock and Extreme Elevation of Procalcitonin Related to Kratom Use. Indian J Crit Care Med 2019;23(5):239–241.

## INTRODUCTION

Kratom, or *Mitrangyna speciosa*, is a plant native to Southeast Asia. It has been used as an herbal remedy for years in rural areas of Malaysia and Thailand for fatigue and pain relief.^1^ Over the past few years, kratom has become increasingly popular in North America as an opioid analgesic alternative, marketed via internet for purchase.^2^ With this increased availability and use, there have been reports regarding its abuse potential and toxicity. The pathobiology effects and clinical management still pose a challenge to the medical fraternity. We recently came across a young male with refractory hypotension, markedly elevated procalcitonin, acute renal failure, and cholestasis following kratom ingestion.

## CASE HISTORY

An 18-year-old male was seen in the emergency department for complaints of severe nausea, vomiting, weakness, and dizziness of 12 hours duration. In the recent past, he had been troubled by poly-substance abuse, depression, and anxiety requiring admission to rehabilitation center. One week after the discharge from the rehabilitation center and 24 hours prior to hospital admission, patient reported to have taken kratom. Even though he had indulged in taking the substance in past, mailed from Malaysia, he never had experienced any symptoms like these. His prescription medications included buspirone, duloxetine, quetiapine, gabapentin, and propranolol which he took the day before admission. His family history was unremarkable. The night before admission, he began experiencing severe nausea and associated with 15 episodes of vomiting, and several episodes of non-bloody diarrhea. Patient also experienced tremors, dizziness, lightheadedness, and near syncope. He reported no fevers, trauma, skin rash, joint pain or weight loss. On presentation, the blood pressure was 61/28 mm Hg, and pulse 150 beats per minute, and his respiratory rate, temperature, and oxygen saturation were normal. He was awake, alert, and ill-appearing with dry mucosal surfaces. The remainders of the examinations were normal. The laboratory results are shown in Table 1. His procalcitonin level was reported to be greater than 200 ng/mL (normal cut off <0.5 ng/mL). Patient received aggressive intravenous fluid administration, and with the suspicion of possible sepsis from unknown source, empiric antimicrobials were initiated. Because of persistent hypotension, vasopressin was started through a central venous catheter. Work up for source of infections such as urinalysis, blood cultures, and imaging (chest X-rays and transthoracic echocardiogram) was found to be negative. After 72 hours of supportive therapy, shock was resolved, and both the renal and liver functions abnormalities started to improve. Empiric antimicrobials were discontinued, and patient was transferred to floor. His subsequent hospital course remained uneventful, and he was discharged with home medication regimen and psychiatry follow-up.

### DISCUSSION

Kratom (*Mitragyna speciose*) has been used in Southeast Asia for decades for its psychoactive properties. It is now being used in the United States for similar purposes.^3^ The key primary alkaloid in kratom is mitragynine, and its effects depend on the dosage.^4^ At low doses, it provides a psychostimulant like effect, while at high doses, it acts like an opioid with analgesic properties. In animal studies, the chemically active components of kratom elicit agonistic activity on µ-opioid receptors and antagonistic activity on δ-opioid receptors.^5^ Due to its opioid-like effects, it has gained popularity in the United States as an alternative therapy for self-management of chronic pain and opioid withdrawal.^2,6^ According to the CDC, the number of kratom exposure incidences reported across the US has increased 10-fold over the last 5 years.^7^ Yet, there are only a handful of reported cases concerning kratom abuse and toxicity, and its potential adverse effects remain relatively unknown.^3,4,6^

**Table 1:** Laboratory values from admission to discharge day

*Laboratory test*	*Admission*	*2nd day*	*3rd day*	*4th day*	*Discharge*
WBC	23.6 × 10^9^/L	12.1 × 10^9^/L	13.6 × 10^9^/L	13.2 × 10^9^/L	7.7 × 10^9^/L
Ca	6.6 mg/dL	7.5 mg/dL	7.8 mg/dL	8 mg/dL	8.1 mg/dL
Bilirubin Total	3.7 mg/dL	3.0 mg/dL	4.5 mg/dL	5.4 mg/dL	2.7 mg/dL
Bilirubin (Direct)	3.2 mg/dL	2.5 mg/dL	3.9 mg/dL	4.8 mg/dL	1.7 mg/dL
Creatinine	4 mg/dL	1.9 mg/dL	1.2 mg/dL	0.9 mg/dL	0.9 mg/dL
AP	96 U/L	127 U/L	192 U/L	302 U/L	353 U/L
ALT	269 U/L	180 U/L	131 U/L	110 U/L	95 U/L
AST	257 U/L	140 U/L	102 U/L	77 U/L	70 U/L
Procalcitonin	>200 ng/dL				9.55 ng/dL

*WBC*: White blood cells; *Ca*: Calcium; *AP*: Alkaline phosphatase; *ALT*: Alanine transferase; *AST*: Aspartate aminotransferase

There have been reports of kratom use being linked to hepatotoxicity, seizures, coma, and in some cases, death.^8–12^ Regular kratom use for 2 weeks and longer has been associated with acute cholestasis.^8,9^ Kratom co-ingestion with other drugs, such as benzodiazepines, o-desmethlytramadol, propylhexedrine, and antidepressants has been associated with significant morbidity and mortality.^6,11,13^ There is still limited information regarding the pharmacology of kratom and its co-interaction with other substances to understand the potential outcomes and adverse effects.

The case described here is unique in two particular findings. To the best of our knowledge, kratom withdrawal or toxicity has not been previously associated with the severe shock and extremely high procalcitonin level like our patients. The commonly reported symptoms reported for kratom withdrawal include nausea, vomiting, myalgias, diarrhea, insomnia, headaches, fever, and tremors.^1,3^ Withdrawal symptoms typically last less than a week and are more severe in chronic users. Our patient's presentation raised concerns for withdrawal/toxicity of kratom, severe hypovolemia, anaphylaxis as well as an infectious etiology leading to sepsis. However, all pertinent investigations were negative for any identifiable infectious source and organism. The etiology of these symptoms was unclear. Procalcitonin has been used to diagnose, and monitor the effectiveness therapy for the myriads of infectious disease process leading to septic shock.^14^ It has high sensitivity and specificity to differentiate infections from other etiology responsible for systemic inflammatory response syndrome.^15^ There has been at least one case reported with elevated procalcitonin (>1000 ng/mL) in the absence of bacterial infection that was associated with amphetamine toxicity.^16^ However, our patient neither had severe infection nor had any history of mechanical injury, burns, pancreatitis, heat stroke, and amphetamine intoxication to explain the high procalcitonin level. We attribute that this patient presentation is related to kratom use, in view of the chronological sequence of events leading to his hospitalization.

However, there was no confirmatory laboratory test performed for the presence of kratom or mitragynine in the serum or urine. Testing for mitragynine is not part of the standard urine toxicology report at our institution, and no follow-up test was performed to quantify mitragynine levels.

### CONCLUSION

Kratom, an herbal remedy used as opioid alternatives, is a growing concern in different parts of the world. Physicians should be vigilant about the potential side effects of the use for appropriate care of the patients.

## References

[1] Singh D,, Muller CP,, Vicknasingam BK. (2014;). Kratom (Mitragyna speciosa) dependence, withdrawal symptoms and craving in regular users.. Drug Alcohol Depend..

[2] Boyer EW,, Babu KM,, Adkins JE,, McCurdy CR,, Halpern JH. (2008;). Self-treatment of opioid withdrawal using kratom (Mitragynia speciosa korth).. Addiction..

[3] Cinosi E,, Martinotti G,, Simonato P,, Singh D,, Demetrovics Z,, Roman-Urrestarazu A, (2015;). Following “the roots” of kratom (Mitragyna speciosa): the evolution of an enhancer from a traditional use to increase work and productivity in southeast Asia to a recreational psychoactive drug in western countries.. BioMed Res Int..

[4] Prozialeck WC. (2016;). Update on the pharmacology and legal status of kratom.. J Am Osteopath Assoc..

[5] Varadi A,, Marrone GF,, Palmer TC,, Narayan A,, Szabo MR,, Le Rouzic V, (2016;). Mitragynine/corynantheidine pseudoindoxyls as opioid analgesics with mu agonism and delta antagonism, which do not recruit beta-arrestin-2.. J Med Chem..

[6] Galbis-Reig D. (2016;). A case report of kratom addiction and withdrawal.. WMJ..

[7] Anwar M,, Law R,, Schier J. (2016;). Notes from the field. kratom (Mitragyna speciosa) exposures reported to poison centers — United States, 2010–2015.. MMWR Morb Mortal Wkly Rep..

[8] Dorman C,, Wong M,, Khan A. (2015;). Cholestatic hepatitis from prolonged kratom use: a case report.. Hepatology..

[9] Kapp FG,, Maurer HH,, Auwarter V,, Winkelmann M,, Hermanns-Clausen M. (2011;). Intrahepatic cholestasis following abuse of powdered kratom (Mitragyna speciosa).. J Med Toxicol..

[10] Nelsen JL,, Lapoint J,, Hodgman MJ,, Aldous KM. (2010;). Seizure and coma following Kratom (Mitragynina speciosa Korth) exposure.. J Med Toxicol..

[11] McIntyre IM,, Trochta A,, Stolberg S,, Campman SC. (2015;). Mitragynine ‘kratom’ related fatality: a case report with postmortem concentrations.. J Anal Toxicol..

[12] Karinen R,, Fosen JT,, Rogde S,, Vindenes V. (2014;). An accidental poisoning with mitragynine.. Forensic Sci Int..

[13] Holler JM,, Vorce SP,, McDonough-Bender PC,, Magluilo J,, Solomon CJ,, Levine B. (2011;). A drug toxicity death involving propylhexedrine and mitragynine.. J Anal Toxicol..

[14] Becker KL,, Snider R,, Nylen ES. (2008;). Procalcitonin assay in systemic inflammation, infection, and sepsis: clinical utility and limitations.. Crit Care Med..

[15] Wacker C,, Prkno A,, Brunkhorst FM,, Schlattmann P. (2013;). Procalcitonin as a diagnostic marker for sepsis: a systematic review and meta-analysis.. Lancet Infect Dis..

[16] Lovas A,, Ágoston Z,, Késmárky K,, Hankovszky P,, Molnár Z. (2014;). Extreme procalcitonin elevation without proven bacterial infection related to amphetamine abuse.. Case Rep Crit Care..

